# Comparison of gene expression profiles altered by comfrey and riddelliine in rat liver

**DOI:** 10.1186/1471-2105-8-S7-S22

**Published:** 2007-11-01

**Authors:** Lei Guo, Nan Mei, Stacey Dial, James Fuscoe, Tao Chen

**Affiliations:** 1 Division of Systems Toxicology, National Center for Toxicological Research, FDA, Jefferson, AR 72079, USA; 2 Division of Genetic and Reproductive Toxicology, National Center for Toxicological Research, FDA, Jefferson, AR 72079, USA

## Abstract

**Background:**

Comfrey (*Symphytum officinale*) is a perennial plant and has been consumed by humans as a vegetable, a tea and an herbal medicine for more than 2000 years. It, however, is hepatotoxic and carcinogenic in experimental animals and hepatotoxic in humans. Pyrrolizidine alkaloids (PAs) exist in many plants and many of them cause liver toxicity and/or cancer in humans and experimental animals. In our previous study, we found that the mutagenicity of comfrey was associated with the PAs contained in the plant. Therefore, we suggest that carcinogenicity of comfrey result from those PAs. To confirm our hypothesis, we compared the expression of genes and processes of biological functions that were altered by comfrey (mixture of the plant with PAs) and riddelliine (a prototype of carcinogenic PA) in rat liver for carcinogenesis in this study.

**Results:**

Groups of 6 Big Blue Fisher 344 rats were treated with riddelliine at 1 mg/kg body weight by gavage five times a week for 12 weeks or fed a diet containing 8% comfrey root for 12 weeks. Animals were sacrificed one day after the last treatment and the livers were isolated for gene expression analysis. The gene expressions were investigated using Applied Biosystems Rat Whole Genome Survey Microarrays and the biological functions were analyzed with Ingenuity Analysis Pathway software. Although there were large differences between the significant genes and between the biological processes that were altered by comfrey and riddelliine, there were a number of common genes and function processes that were related to carcinogenesis. There was a strong correlation between the two treatments for fold-change alterations in expression of drug metabolizing and cancer-related genes.

**Conclusion:**

Our results suggest that the carcinogenesis-related gene expression patterns resulting from the treatments of comfrey and riddelliine are very similar, and PAs contained in comfrey are the main active components responsible for carcinogenicity of the plant.

## Background

Comfrey (*Symphytum officinale*) is consumed by humans as a vegetable and a tea. It has been used as an herbal medicine for more than 2000 years to treat broken bones, tendon damage, ulcerations in the gastrointestinal tract, lung congestion, and joint inflammation, and to promote wound healing [1]. It, however, has been reported that comfrey is hepatotoxic in livestock and humans, and carcinogenic in experimental animals. Comfrey induced hepatic veno-occlusive lesion (VOD) in humans [2-4] and hepatocellular adenomas and hemangioendothelial sarcomas in rats [5]. Therefore, the regular use of comfrey is a potential health risk for development of liver cancers. In 2001, the US Food and Drug Administration requested voluntary compliance for the removal of products containing comfrey [6].

It is still not clear about the mechanism of tumor induction by comfrey because comfrey is a mixture of many different substances and the active components responsible for the carcinogenesis have not been identified. It has been suggested that the induction of hepatic tumors has been associated with the pyrrolizidine alkaloids (PAs) that are present in comfrey [7-9] since PAs are genotoxic and carcinogenic in liver [10,11]. Recently, we demonstrated that the PAs in the comfrey plant appear to be responsible for mutation induction in rat liver [12]. Mutations are involved in the etiology of cancer [13]. Research results in molecular cancer genetics have identified inherited and somatic cell mutations associated with cancer in oncogenes, tumor suppressor genes, DNA repair genes and other related genes [14-17]. Therefore, we hypothesize that PAs contained in comfrey are the main active components resulting in tumors in liver.

Riddelliine is one of the tumorigenic PAs and has been studied as a prototype of PA. The toxicity and carcinogenicity of riddelliine have been studied by the National Toxicological Program (NTP) [18-20], and the mechanism of riddelliine-induced tumorigenicity in experimental animals has been studied at the National Center for Toxicological Research (NCTR) [21-27]. Results showed that (1) riddelliine was metabolized to the major metabolites 6,7-dihydro-1-hydroxymethyl-5*H*-pyrrolizine (DHP) and riddelliine *N*-oxide; (2) DHP-derived DNA adducts were formed both in vivo and in vitro; (3) G:C → T:A transversions were the major type of mutation induced in the liver of riddelliine-treated rats; and (4) riddelliine caused liver tumors in male mice and both sexes of rats, mononuclear cell leukemia in rats, and lung neoplasms in female mice.

DNA microarray, a key advanced technology, has developed rapidly because of its ability to examine the expression levels of thousands of genes simultaneously. It is used increasingly for identifying biomarkers, elucidating patterns of gene expression, and understanding the mechanism of disease and toxicity. Toxicogenomics applies high through-put genomics tools to the study of toxicology and gene expression microarrays have been used extensively [28]. The interpretation of gene expression data, however, can be complicated by the gene expression alterations caused by environmental factors such as diet [29] and time of day [30,31]. That is, it can be difficult to discern toxin-specific gene expression changes from those due to environmental effects. This can be complicated further when the test article is a complex food such as comfrey.

To examine the possible PA-induced affects on gene expression caused by comfrey, the gene expression profiles in the livers of comfrey-treated rats were compared to the gene expression profiles from rats treated with the purified PA riddelliine. The correlation of the gene expression and biological functions related to carcinogenesis between the two treatments were explored.

## Results and discussion

Previously, we investigated the mutagenicity of comfrey and riddelliine in rats [26,32] under conditions which have been shown to result in liver tumors [5,20]. In the original mutation studies, 8% comfrey treatment induced about a 4-fold higher mutant frequency (MF) over the control group [32], while 1 mg/kg riddelliine exposure resulted in about a 3-fold increase in MF compared to controls [26]. In these studies, the rats were exposed to the compounds for 12 weeks and then sacrificed. No tumors were visible at this time in our study, although Hirono et al. reported that liver tumors developed in the rats as early as 7 weeks after initiation of feeding comfrey [5]. The mutation spectra for comfrey- and riddelliine-treated rats were significantly different from the controls, but there was no significant difference between the spectra for comfrey and riddelliine. G:C → T:A transversion (35–42%) was the major type of mutation in both comfrey and riddelliine treated rats, with 8–17% tandem base substitutions [12]. These mutational data from comfrey-treated rats suggests that PAs in the plant are responsible for mutation induction and tumor initiation in rat liver.

To further investigate the effects of comfrey and riddelliine on gene expression changes in liver, microarray analysis was performed on liver samples from these high-dose groups. The details of each experiment and gene expression analysis were reported previously [32,33]. In the present study, gene expression alterations caused by exposure to comfrey were compared to those caused by exposure to the representative PA riddelliine.

### Comparison of gene expression profiles induced by comfrey and riddelliine

**Figure 1:**
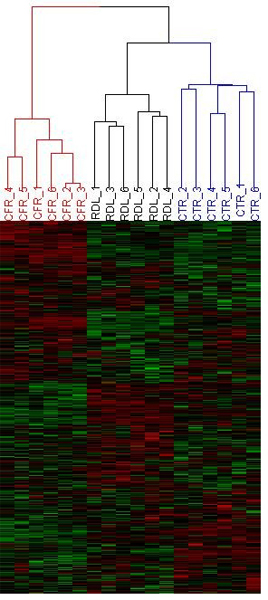
**Hierarchical Cluster Analysis (HCA) of expression profiles for control, 8% comfrey-fed, and 1 mg/kg riddelliine treated groups**. The log_2 _intensity of the entire gene set was scaled by *Z*-score transformation, and then these values were hierarchically clustered using Euclidean distance metric and average linkage. Each column represents the results from an individual animal. CTR, control; CFY, comfrey treatment; RDL, riddelliine treatment.

**Figure 2:**
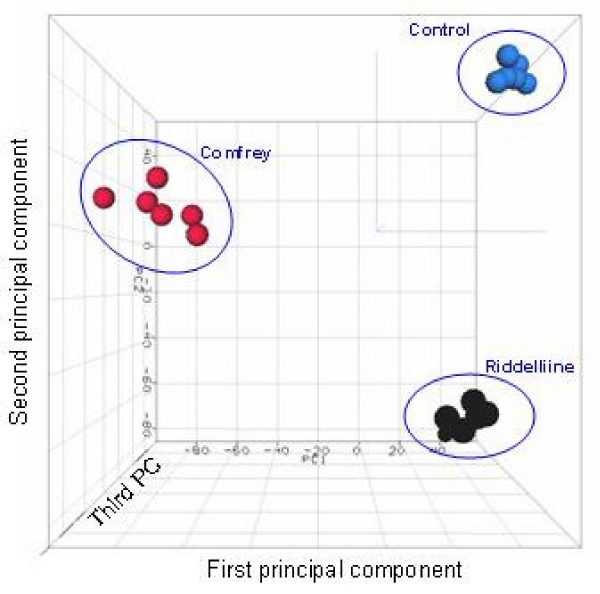
**Principal Component Analysis (PCA) of expression profiles for control, 8% comfrey-fed, and 1 mg/kg riddelliine treated groups**. The intensity of the entire gene set was used, and no specific cut off was applied.

The intensities of the whole rat gene data were normalized by quantile normalization and then analyzed by Hierarchical Cluster Analysis (HCA) and Principal Component Analysis (PCA) to visualize clusters of samples corresponding to the different treatments (Figures 1 and 2). The result of the HCA of the gene expression data is shown in Figure 1, and shows that samples were grouped together according to the treatments. There were clear separations between of the control, comfrey-treated, and riddelliine-treated groups. The gene expression pattern due to comfrey exposure was distinct from that due to riddelliine exposure (Figure 2), possibly reflecting the effects of many other substances in comfrey other than PAs [9]. This difference is expected to be the result of different properties of two chemicals including pharmacological and toxicological effects.

**Figure 3:**
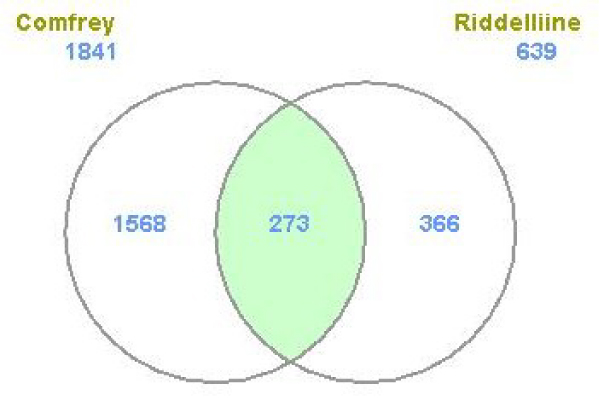
**Numbers of differentially expressed genes (DGEs) regulated by riddelliine and comfrey treatment**. A gene was identified as differentially expressed if the fold-change was greater than 2 (up or down) and the *P*-value was less than 0.01 in comparison to the control group. The color in green refers to the number of genes whose expressions were significantly altered by both comfrey and riddelliine.

The differentially expressed genes between the treatment and control groups were identified based on simple *t*-test. The criteria used to classify a gene as differentially expressed were that there was at least a two-fold change in the gene expression compared to the controls and a *P*-value less than 0.01 for the fold-change difference. By locus link ID, a total of 1814 genes from the comfrey treatment and 639 genes from the riddelliine treatment satisfied the requirements; both with about equal numbers of up- and down-regulated genes in response to the treatment [32,33]. The number of genes whose expression was altered by comfrey was much higher than that by riddelliine, possibly due to the fact that comfrey is a complex mixture of biologically active compounds. By locus link ID, there were 302 genes that were common between comfrey and riddelliine; the majority of these common genes (90%, 273 genes) were regulated in the same direction (Figure 3). Since PAs generally produce the same types of DNA adducts [10] and both comfrey and riddelliine have similar mutational spectra [12,32], it would be expected that the common genes altered by riddelliine and comfrey in this study may contribute to PA-induced toxicity.

### Common drug metabolizing genes induced by comfrey and riddelliine

A striking feature of the biotransformation enzymes is that their activities can be induced following exposure of chemicals or drugs. Therefore, examination of drug metabolizing genes (DMGs) can provide important pharmacological information, with potential clinical and toxicity implications for a given chemical. Liver is the major organ for biotransformation of xenobiotics and drugs, and PAs require metabolic activation to exert their biological and toxicological effects. Study and comparison of these genes altered by comfrey and riddelliine can provide us valuable information about common mechanisms on PAs' biotransformation and metabolisms. Also, in these steps PAs are activated into carcinogen, initiating the carcinogenesis. Therefore, we focused our investigation on the expression changes of DMGs. There were 45 and 87 DMGs up- or down-regulated by the treatment of riddelliine and comfrey, when a cut off of 2-fold change and *P *< 0.01 were used as the criteria to select genes. The expressions of 22 of these genes were altered by both comfrey and riddelliine, and are detailed in Table 1. The changed expression of each gene was always in the same direction for the 2 compounds. These commonly regulated DMGs were grouped into the three drug metabolism phases (phase I, II and III).

**Table 1:** Genes involved in drug metabolism altered by comfrey and riddelliine treatments in liver

**Gene symbol**	**Gene description**	**Locus link ID**	**Fold change^a^**	
			
			**Comfrey**	**Riddelliine**
*Phase I metabolism*				
Ces2	carboxylesterase 2 (intestine, liver)	171118	10.4	2.4
Cyp2c	cytochrome P450, subfamily IIC	29277	-24.3	-270.3
Cyp2c40	cytochrome P450, family 2, subfamily c	25011	8.7	42.3
Cyp2e1	cytochrome P450, family 2, subfamily e	25086	2.2	2.1
Fmo5	flavin containing monooxygenase 5	246248	4.1	4.2
*Phase II metabolism*				
Gsta3	glutathione S-transferase, alpha 3	14859	22.5	13.4
Inmt	indolethylamine N-methyltransferase	21743	-29.3	-11.7
Nqo1	NAD(P)H dehydrogenase, quinone 1	24314	5.0	2.6
Sult1c1	sulfotransferase family, cytosolic,1C, member1	65185	-2.7	-3.2
*Phase III metabolism*				
Abcb1	ATP-binding cassette, sub-family B	24646	97.0	11.3
Abcb9	ATP-binding cassette, sub-family B, member 9	63886	-5.0	-2.7
Abcc3	ATP-binding cassette, sub-family C, member 3	140668	25.1	10.8
Abcc8	ATP-binding cassette, sub-family C, member 8	25559	-9.7	-2.6
Atp13a5	ATPase type 13A5	268878	-3.6	-4.1
Slc13a5	solute carrier family 13, member 5	266998	-2.9	-2.4
Slc16a4	solute carrier family 16, member 4	229699	-2.8	-4.9
Slc22a6	solute carrier family 22, member 6	29509	-3.7	-2.4
Slc22a8	solute carrier family 22, member 8	83500	-12.2	-833.3
Slc25a21	solute carrier family 25, member 21	171151	4.4	2.7
Slc25a30	solute carrier family 25, member 30	67554	-4.5	-2.7

^a^The symbol of minus (-) means down-regulation, and the *P*-value for all genes is less than 0.01.

There are postulated to be three phase 1 pathways for the metabolism of PA; (1) Oxidation via cytochrome P450 (Cyp), (2) *N*-oxidation via flavin-containing monooxygenases (Fmo), and (3) hydrolysis via carboxylesterases. Hepatic cytochrome P450, Cyp3a and Cyp2b isoforms, were the major metabolizing enzymes involved in PA metabolism in humans [10]. One gene of the Cyp2b family (*Cyp2b15*) and three genes of the Cyp3a family (*Cyp3a2*, *3a9 *and *3a18*) were contained on the microarray. Although riddelliine elevated the level of *Cyp3a9 *RNA, no significant changes in the expression of these genes was induced by both riddelliine and comfrey exposure in our study. However, a significant induction of *Cyp2c12 *(*Cyp2c40*) was found with the expression increased by 8.7- and 42-fold by the treatments with comfrey and riddelliine, respectively (Table 1). This suggests that Cyp2c may play an important role in metabolizing comfrey and riddelliine in addition to Cyp3a and 2b.

Flavin-containing monooxygenases were reported to be involved in the biotransformation of PAs to the *N*-oxide metabolites [34], and *N*-oxidation appears to be one of the crucial pathways in determining the toxicity of PAs. However, it is not well known which particular family member contributes to this metabolism and toxicity. Among five flavin-containing monooxygenase genes (Fmo1-5) contained on microarray, *Fmo5 *was induced about 4-fold by both comfrey and riddelliine treatments. Hydrolysis of PAs is considered to be a major detoxification pathway in phase I. As shown in Table 1, *carboxylesterase 2 *(*Ces2*) was induced by the treatments of comfrey and riddelliine in our study. Our results not only confirm the findings reported in the literature but also provide additional detail that Fmo5 may be the form of flavin-containing monooxygenases family involved in *N*-oxidation and carboxylesterase 2 may play a role in hydrolysis of these chemicals.

Phase II and phase III enzymes usually function as detoxication. Phase II enzymes involve in conjugation of the polar functional groups of phase I metabolites and phase III enzymes are transporters for these metabolites. In general, exposure to some xenobiotics can trigger cellular "stress" response leading to an increase in the gene expression of phases II and III, which ultimately enhances the elimination and clearance of these xenobiotics. Treatment with comfrey and riddelliine has been shown to alter the expression of a number of phases II and III genes along with phase I genes (Table 1). The expression of the *glutathione S-transferase*, *alpha 3 *(*Gsta3*) gene increased 22- and 13-fold over the control with comfrey and riddelliine treatment, respectively. Glutathione S-transferases are major phase II detoxification enzymes found mainly in the cytosol. It has been reported that PAs can be conjugated with glutathione by these enzymes, reduce glutathione concentrations in liver, and increase the activity of these conjugation enzymes after PAs treatment [35-37]. This result is consistent with those previously studied. In the phase III genes, *ATP-binding cassette*, *sub-family B *(*Abcb1*) was induced by comfrey and riddelliine 97- and 11-fold over the control, respectively. No publications have been found about the relationship between PA treatment and induction of ATP-binding cassette. Future research is encouraged for the role of ATP-binding cassette in PAs' metabolism and carcinogenesis.

**Figure 4:**
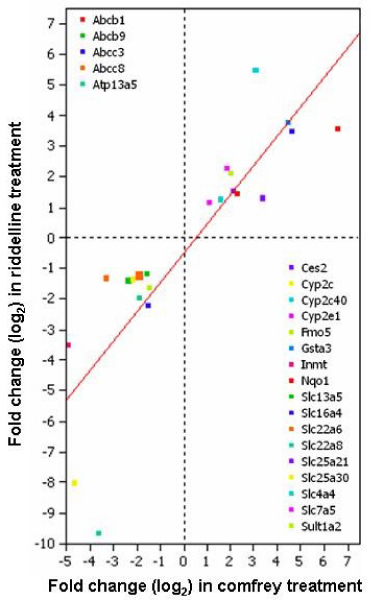
**Comparison of fold-change of drug metabolizing genes (DMG) whose expression was altered by both comfrey and riddelliine treatments**. Twenty-two DMGs detailed in Table 1 were commonly regulated by comfrey and riddelliine.

Not surprisingly, there were more DMGs altered by comfrey treatment than those by riddelliine treatment. This may be because comfrey is a mixture of many substances in addition to PAs, and these other substances may be involved in the induction of other DMGs. Nearly 50% of DMGs altered by the treatment with riddelliine were also found altered by the treatment with comfrey. It should be note that all of these 22 DMGs regulated by both chemicals changed in the same direction (Table 1). The gene expression similarity between the two treatments was further assessed by calculating the correlation coefficients of the log_2 _fold changes. The result is displayed as a scatter plot in Figure 4. The correlation coefficient was 0.72, indicating a fairly good agreement between the two treatments and suggesting that the toxicity of comfrey results from activation of PA. Although the process of drug metabolism is complicated, our results at least indicate that there is a common mechanism of drug metabolism involved in these two chemical treatments.

### Common biological processes and genes associated with carcinogenesis

**Figure 5:**
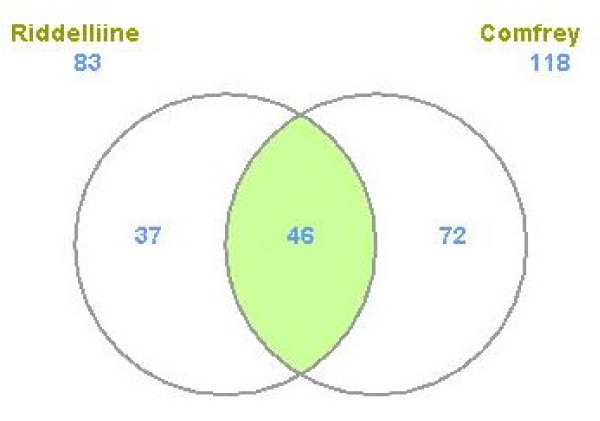
**Number of regulated function processes significantly altered (*P *< 0.01) by comfrey and riddelliine treatments**. The color in green refers to the number of function processes that were significantly altered by both riddelliine and comfrey.

The Ingenuity Pathway Analysis Knowledge database, which provides a classification of gene products into molecular functions, biological processes, and cellular components, was used to help understand the biological consequences of exposure to comfrey or riddelliine. Biological process was examined for the genes from comfrey and riddelliine individually, and the software placed the processes into functional processes or categories such as cancer and cell death. After a cut off of *P *< 0.01, we observed that there were 83 and 118 functional processes for riddelliine and comfrey treatments, respectively. Among them, 46 of the function processes were altered by both comfrey and riddelliine, which was about half of the function processes altered by riddelliine and one-third of the function processes altered by comfrey (Figure 5). The top categories for these functions are listed in Table 2, including cancer, cell death, cell morphology, cell-to-cell signaling and interaction, and tissue development. These results suggest that common mechanism(s) may be responsible for the toxicity of both comfrey and riddelliine.

**Table 2:** The major relevant functions altered by both comfrey and riddelliine treatments in liver

**Function category**	**Number of processes**
Cancer	35
Cell death	35
Small molecule biochemistry	17
Cell morphology	14
Cell-to-cell signaling and interaction	14
Lipid metabolism	12
Molecular transport	12
Tissue development	11
Cellular movement	10

**Figure 6:**
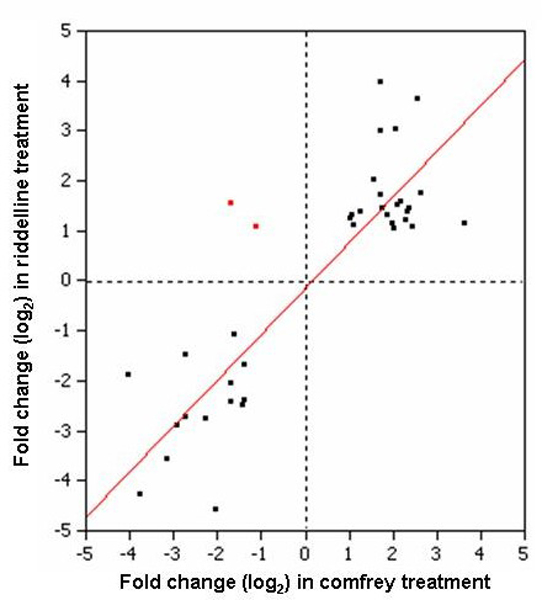
**Comparison of fold-change of cancer-related genes whose expression was altered by both comfrey and riddelliine treatments**. The genes commonly regulated by comfrey and riddelliine are listed in Table 3. Black symbol means regulated in same direction, and red symbol means regulated in opposite direction.

The finding that both comfrey and riddelliine treatments trigger gene expression alterations associated with cancer and cell death processes in this study is likely due to the PAs. As discussed above, PAs produce hepatotoxicity and carcinogenesis in experimental animals and humans. Functional analysis with Ingenuity Pathway Analysis showed that comfrey which contains 9 PAs [7-9] altered many more genes involved in cancer related pathways than riddelliine. The expressions of 387 cancer-related genes were significantly altered after comfrey exposure and the expressions of 84 cancer-related genes were significantly changed by riddelliine treatment. These genes are involved in the many functions associated with different stages of carcinogenesis. A large number of genes related to both treatments may indicate the PAs' carcinogenic insults. Table 3 shows the detailed information of 42 genes whose expressions were significantly altered by both riddelliine and comfrey treatments. These common cancer-related genes include genes involved in apoptosis and cell death, invasion, cell growth, cell morphology, and cell cycle. Figure 6 shows the strong correlation between the log_2 _fold-changes in gene expression caused by the two treatments; only two of the genes were regulated in the opposite directions. These results also indicate that the carcinogenicity of comfrey is generated from PAs.

**Table 3:** Genes involved in carcinogenesis altered by comfrey and riddelliine treatments in liver

**Gene symbol**	**Gene description**	**Locus link ID**	**Fold change^a^**	
			
			**Comfrey**	**Riddelliine**
AKR1C3	aldo-keto reductase family 1, member C3	171516	-4.9	-39.5
AR	androgen receptor (dihydrotestosterone receptor)	24208	-13.6	-19.6
ARHGDIG	Rho GDP dissociation inhibitor (GDI) gamma	14570	-8.9	-12.1
C5ORF13	chromosome 5 open reading frame 13	338475	-7.5	-7.6
CAST	Calpastatin (includes EG:831)	25403	-3.3	-5.4
CD36	CD36 molecule (thrombospondin receptor)	29184	3.3	7.9
CDKN1C	cyclin-dependent kinase inhibitor 1C (p57, Kip2)	246060	5.1	2.7
CHEK1	CHK1 checkpoint homolog (S. pombe)	140583	2.1	2.5
CREM	cAMP responsive element modulator	25620	-2.6	-3.3
DOK1	docking protein 1, 62kDa (downstream of tyrosine kinase 1)	13448	4.5	2.9
ECGF1	endothelial cell growth factor 1 (platelet-derived)	72962	-6.7	-2.8
EGR1	early growth response 1	24330	12.2	2.2
FABP2	fatty acid binding protein 2	25598	3.3	2.7
FAS	Fas (TNF receptor superfamily, member 6)	246097	3.3	3.2
FEZ1	fasciculation and elongation protein zeta 1 (zygin I)	81730	*-3.2*	*2.9*
FUT1	fucosyltransferase 1	81919	-4.8	-6.9
FYN	FYN oncogene related to SRC, FGR, YES	25150	4.3	2.8
GDF15	growth differentiation factor 15	29455	4.8	2.3
HGF	hepatocyte growth factor (hepapoietin A; scatter factor)	24446	3.6	2.4
HRASLS3	HRAS-like suppressor 3	24913	3.0	4.0
HSPA1A	heat shock 70kDa protein 1A	3303	4.1	8.1
ID1	inhibitor of DNA binding 1,	25261	3.9	2.2
IGFBP2	insulin-like growth factor binding protein 2, 36kDa	25662	3.2	15.7
ITGA4	integrin, alpha 4 (antigen CD49D)	16401	5.4	2.1
LAMA5	laminin, alpha 5	3911	37.5	2.1
LIMK2	LIM domain kinase 2	29524	4.1	2.0
MGAT5	mannoside acetylglucosaminyltransferase 5	65271	*-2.2*	*2.1*
MT1A	metallothionein 1A (functional)	24567	5.8	12.2
NCR1	natural cytotoxicity triggering receptor 1	117547	2.5	61.7
NQO1	NAD(P)H dehydrogenase, quinone 1	24314	5.0	2.6
PPARG	peroxisome proliferative activated receptor, gamma	25664	-2.6	-5.3
PRLR	prolactin receptor	24684	29.8	284.4
PTK6	PTK6 protein tyrosine kinase 6	20459	-16.7	-3.7
RTN4	reticulon 4	83765	6.2	3.4
SERPINA5	serpin peptidase inhibitor, clade A, member 5	65051	-3.1	-2.2
SMOX	spermine oxidase	228608	2.0	2.3
ST6GAL1	ST6 beta-galactosamide alpha-2,6-sialyltranferase 1	20440	-2.7	-5.7
ST8SIA1	ST8 alpha-N-acetyl-neuraminide alpha-2,8-sialyltransferase 1	25280	-6.8	-6.8
TIMP3	TIMP metallopeptidase inhibitor 3	25358	2.1	2.1
TNF	tumor necrosis factor (TNF superfamily, member 2)	24835	-4.2	-24.5
TNFSF10	tumor necrosis factor (ligand) superfamily, member 10	246775	2.4	2.6
TUBG1	tubulin, gamma 1	252921	-3.2	-4.2

^a^The symbol of minus (-) means down-regulation, and the *P*-value for all genes is less than 0.01.

Most of genes involved in apoptosis (*Cdkn1c*, *Egr1*, *Fas*, *Gdf15*, *Hgf*, *Hrasls3*, *Hspa1a*, *Lama5*, *Smox*, *Tnfsf10*) were up-regulated and may be explained by the removal of cells damaged by PAs. For example, *Egr1 *(early growth response 1), whose expression increased 12- and 2-fold over the control with comfrey and riddelliine treatments, respectively, has the ability to function in numerous capacities, including differentiation, growth, growth inhibition, and apoptosis depending on the cell type and the stimulus. In response to stress, *Egr1 *displays a remarkable functional similarity to *p53 *and *p73 *[38] and may be involved in protection of the cell against the toxicity of these compounds. We also observed that comfrey and riddelliine significantly up-regulated Hspa1a, the gene encoding heat shock protein (HSP) 70 family members. High levels of inducible HSP70s prevent stress-induced apoptosis and block caspase activity, mitochondrial damage, and nuclear fragmentation [39]. These genes may all be involved in repair and protection of the cell against the damage caused by the PAs.

Among the common cancer-related genes, the expressions of two genes (*Fez1 *and *Mgat5*) were altered in opposite directions (down-regulated by comfrey and up-regulated by riddelliine). *Mgat5 *encodes N-acetylglucosaminyltransferase V (GlcNAc-TV), the Golgi enzyme required in the N-glycan processing pathway that modifies glycoproteins, including the cytokine receptors. *Mgat5 *gene expression is up-regulated by Ras pathway activation [40]. In *Mgat5*^-/- ^mice, tumor latency is longer and metastasis is reduced compared with *Mgat5*^+/+ ^mice [41]. FEZ1 (Fasciculation and elongation protein zeta-1) is a tumor suppressor gene that maps to chromosome 8p22, a chromosomal region frequently deleted in many human malignancies. The FEZ1 gene is expressed almost ubiquitously in normal tissues, and is prone to inactivation in human cancer [42]. In addition, introduction of FEZ1 into Fez1-negative cancer cells results in suppression of tumorigenicity and reduced cell growth with accumulation of cells at late S-G_2_/M stage of the cell cycle [43]. These two genes appear to be important in the carcinogenesis process and the differences in their expression in the two treatment groups may be an indication that the livers in the two groups are in different stages of the carcinogenic process. Alternatively, this may reflect the contribution of other components in comfrey.

## Conclusion

Previously we found that the mutagenicity of comfrey was associated with the PAs contained in the plant. Therefore, we suggested that the toxicity and carcinogenicity of comfrey resulted from these PAs. In this study, we analyzed gene expression profiles in the liver of rats treated with riddelliine, a widely studied genotoxic PA and a proven rodent mutagen and carcinogen, and comfrey, an herbal tea that contains as many as nine PAs. We used the systematic approaches of HCA, PCA, functional analysis, and biological process analysis. Although there were large differences between the genes whose expression was significantly altered by these two treatments, there are common genes and function processes which may be indicative of effects due to PAs. In support of this is the finding that there were strong correlations between the gene expression fold-change alterations caused by the two treatments, in particular, drug metabolizing genes and cancer-related genes. These results suggest that comfrey and riddelliine may share common mechanism(s) of carcinogenesis, and further support our hypothesis that PAs are the main active components for carcinogenesis of comfrey.

## Materials and methods

### Treatments with comfrey and riddelliine

The details on the description of the *in vivo *portion of this study has been described previously [26,32]. Briefly, Big Blue Fisher 344 transgenic rats were fed with a diet containing 8% comfrey root for 12 weeks or gavaged with 1 mg/kg riddelliine 5 times a week for 12 weeks. One day after the last treatment, 6 rats from each vehicle control, comfrey-treated, and riddelliine-treated groups were sacrificed, and the livers were isolated and stored at -80°C for mutagenesis and microarray analysis. Big Blue transgenic rats were obtained from Taconic Laboratories (Germantown, NY) through purchase from Stratagene (La Jolla, CA). All animal procedures followed the recommendations of the NCTR Institutional Animal Care and Use Committee for the handling, maintenance, treatment, and sacrifice of the rats.

### Microarray processing

The detailed description of the microarray processing of this study has been reported previously [32,33]. Briefly, total RNA was isolated from liver tissues of 6 control, 6 comfrey-fed, and 6 riddelliine-treated rats using an RNeasy system (Qiagen, Chatsworth, CA). All RNA targets were labeled using the Applied Biosystems RT-IVT Labeling Kit Version 2.0. Digoxigenin labeled cRNA targets were hybridized to Applied Biosystems Rat Whole Genome Survey Microarrays using the Applied Biosystems Chemiluminescent Detection Kit. Images were auto-gridded and the chemiluminescent signals were quantified, corrected for background, and finally, spot- and spatially-normalized using the Applied Biosystems 1700 Chemiluminescent Microarray Analyzer software version 1.1.

### Microarray data analysis

Raw microarray intensity data from the Applied Biosystems' Rat Genome Survey Microarray, which is a one channel microarray with chemiluminescence detection and contains 26,857 probes, were normalized with quantile normalization which is recommended by the manufacturer. The normalized data were then input to ArrayTrack, a software system developed by the NCTR for the management, analysis, visualization and interpretation of microarray data [44]. The identification of differentially expressed genes based on fold-change and *t*-tests cutoffs, and HCA and PCA were conducted within ArrayTrack. Ingenuity Pathway Analysis (Mountain View, CA) was used for network and function analysis.

## Competing interests

The authors declare that they have no competing interests.

## Authors' contributions

All authors were involved in the analysis of microarray data, wrote the manuscript, and approved the final version of manuscript.
